# Impact of Host–Guest Interactions on the Dielectric Properties of MFM-300 Materials

**DOI:** 10.1021/acs.inorgchem.3c02110

**Published:** 2023-10-09

**Authors:** Xi Chen, Sergei Sapchenko, Wanpeng Lu, Ming Li, Meng He, Yinlin Chen, Mark D. Frogley, Ivan da Silva, Sihai Yang, Martin Schröder

**Affiliations:** † Department of Chemistry, University of Manchester, Manchester M13 9PL, U.K.; ‡ Faculty of Engineering, University of Nottingham, Nottingham NG7 2RD, U.K.; § Diamond Light Source, Harwell Science Campus, Oxfordshire OX11 0DE, U.K.; ∥ ISIS Facility, Science and Technology Facilities Council (STFC), Rutherford Appleton Laboratory, Didcot OX11 0QX, U.K.

## Abstract

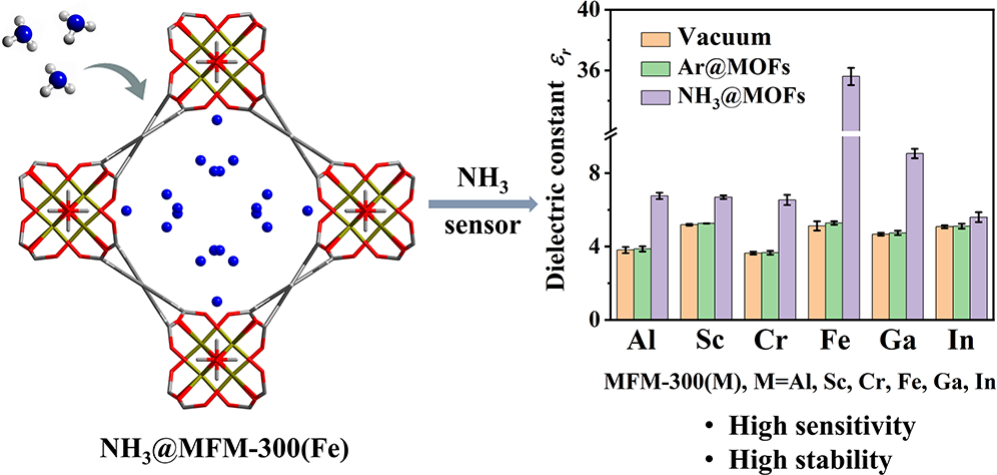

Metal–organic framework (MOF) materials are attracting increasing interest in the field of electronics due to their structural diversity, intrinsic porosity, and designable host–guest interactions. Here, we report the dielectric properties of a series of robust materials, MFM-300(M) (M = Al, Sc, Cr, Fe, Ga, In), when exposed to different guest molecules. MFM-300(Fe) exhibits the most notable increase in dielectric constant to 35.3 ± 0.3 at 10 kHz upon adsorption of NH_3_. Structural analysis suggests that the electron delocalization induced by host–guest interactions between NH_3_ and the MOF host, as confirmed by neutron powder diffraction studies, leads to structural polarization, resulting in a high dielectric constant for NH_3_@MFM-300(Fe). This is further supported by ligand-to-metal charge-transfer transitions observed by solid-state UV/vis spectroscopy. The high detection sensitivity and stability to NH_3_ suggest that MFM-300(Fe) may act as a powerful dielectric-based sensor for NH_3_.

## Abstract 2

Metal-organic framework (MOF) materials are attracting increasing interest in the field of electronics due to their structural diversity, intrinsic porosity, and designable host-guest interactions.

## Introduction

Porous metal–organic framework (MOF) materials show enormous potential for applications in gas storage and separation,^1−3^ substrate binding and delivery,^4^ proton conductivity,^5^ and catalysis^6^ owing to their high porosity and structural diversity. Recently, the dielectric properties of MOFs have been investigated by both theoretical and experimental studies, uncovering the emerging potential of these materials as sensors and gate dielectrics in integrated circuits.^7−11^ For example, a series of IRMOFs with different carboxylate linkers have been identified by theoretical modeling as promising candidates to replace conventional SiO_2_ materials (ε_*r*_ ∼ 4) due to their ultralow dielectric constant (ε_*r*_ < 2).^7,12^ The dielectric properties of HKUST-1 have been investigated as a thin film, bulk pellet, and as a single crystal over a wide range of frequencies (static–kHz–MHz–THz).^13−16^ The static dielectric constant (ε_*r*_) of a thin film of HKUST-1 was estimated to be ε′ = 1.93, where ε′ = *n*^2^ and *n* is the refractive index measured by spectroscopic ellipsometry at a wavelength of 750 nm at 200 °C.^13^ Recently, the dielectric constant of a pellet of desolvated HKUST-1 was measured to be 1.79 at 10 kHz and has been reported to increase to 5.54 upon adsorption of MeOH and water.^14^ The variation of the dielectric constant for a single crystal of HKUST-1 upon loading with guest molecules has been analyzed and gave values for ε_*r*_ of 64, 10.40, 7.51, and 2.95 at 1 MHz for H_2_O-, MeOH-, EtOH-, and I_2_-loaded materials, respectively.^16^ Thus, the porous nature of the MOF affords an excellent platform to vary and control the dielectric property by tuning host–guest interactions, particularly with polar guest molecules. Although the frequency-dependent dielectric constant can be controlled by temperature, the effects of pressure of pelletization, the presence of coordinated or free guest molecules,^7,15−20^ and the impact of host–guest interactions and polarizability on the dielectric constant of MOFs remain rarely explored.^14^

We selected the materials MFM-300(M) {[M_2_(OH)_2_(BPTC)], H_4_BPTC = biphenyl-3,3′,5,5′-tetracarboxylic acid, M = Al, Sc, Cr, Fe, Ga, In}^21−27^ to study the impact of host–guest interactions on their dielectric properties since they show exceptional stability upon adsorption of corrosive gases. Ar and NH_3_ were used as nonpolar and polar probes, respectively. *In situ* AC impedance spectroscopy was employed to evaluate the change in the dielectric constant of bulk pellets of MFM-300 during the adsorption and desorption processes. Adsorption of NH_3_ in MFM-300(Fe) results in the most notable increase in dielectric constant among the MFM-300 series, suggesting a good candidate for dielectric sensing for NH_3_, and the stability and sensitivity of MFM-300(Fe) upon loading of different concentrations of NH_3_ have been studied. Solid-state UV/vis spectroscopy and comparative analysis of the crystal structures of MFM-300(Fe) and MFM-300(Al) upon adsorption of ND_3_ have revealed the presence of host–guest charge transfer in ND_3_@MFM-300(Fe), which leads to the change in the dielectric property.

## Results and Discussion

### Dielectric Measurements

**Figure 1:**
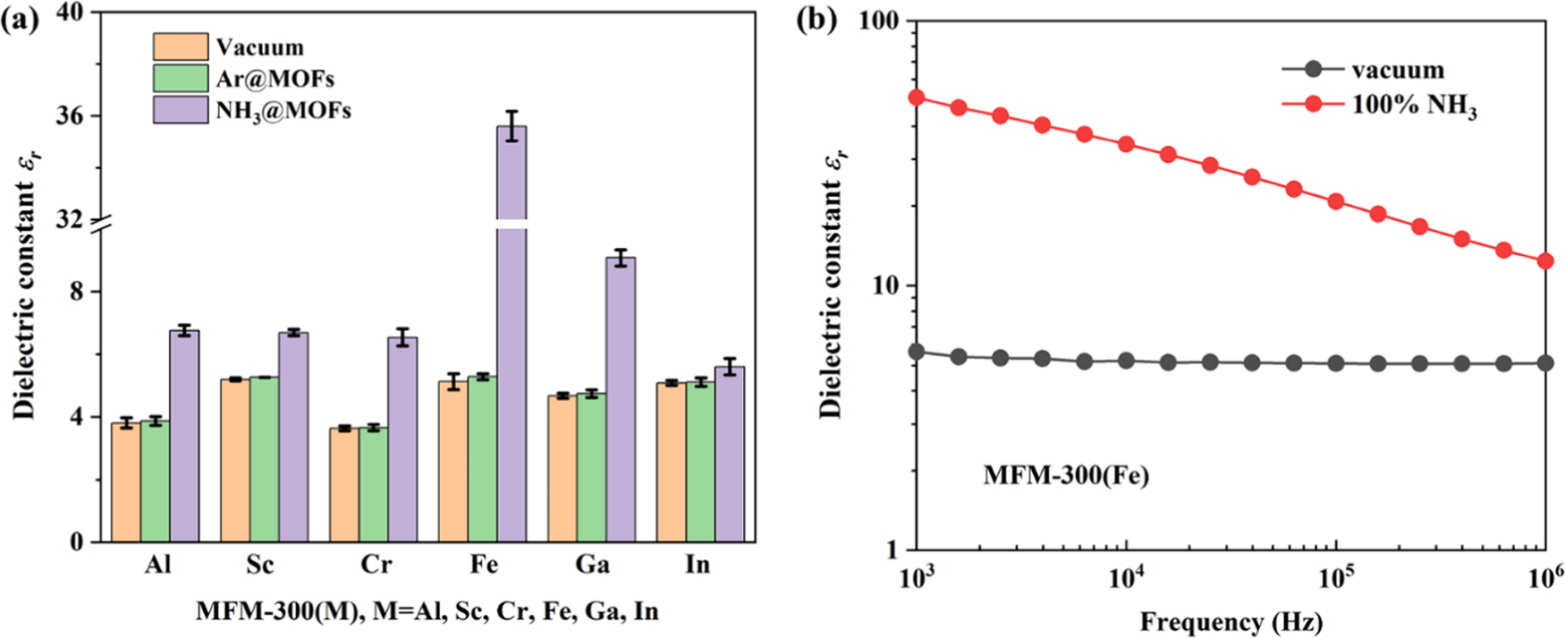
(a) Dielectric constants for MFM-300(M) (M = Al, Sc, Cr, Fe, Ga, In) at 10 kHz under vacuum and upon adsorption of Ar and NH_3_ at 25 °C and 0.5 bar. (b) Frequency-dependent dielectric constant of MFM-300(Fe) before and after NH_3_ adsorption.

The iso-structural materials MFM-300(M) show a 3D framework structure constructed of 1D metal oxide chains [M(OH)_2_O_4_]_∞_ bridged by organic linkers to give square-shaped channels of 6–8 Å diameter along the *c* axis.^22,23^ We sought to monitor the change of the dielectric constant of MFM-300 upon loading of different guest molecules using AC impedance spectroscopy at room temperature. Bulk pellets of MFM-300(M) were placed into an electrochemical gas cell equipped with electrodes (Figure S1), and powder X-ray diffraction (PXRD) analysis confirmed the phase purity of as-synthesized materials (Figure S2). The gas cell was placed under vacuum (1.0 × 10^–2^ mbar) and then loaded with different gases at 0.5 bar (see details in the Supporting Information). Figure 1a shows the dielectric constant ε_*r*_ at 10 kHz for MFM-300(M) (M = Al, Sc, Cr, Fe, Ga, In) under vacuum and upon loading different guest molecules (Tables S1 and S2). Each measurement of the dielectric constant was recorded upon reaching adsorption equilibrium such that the dielectric constant remained constant (Figure S3). Compared with MOFs under vacuum, the Ar-loaded materials show little change of ε_*r*_ since Ar is a nonpolar guest molecule with only weak host–guest interactions. For example, MFM-300(Fe) and Ar@MFM-300(Fe) exhibit values for ε_*r*_ of 5.12 ± 0.25 and 5.28 ± 0.10, respectively, at 10 kHz. By contrast, adsorption of NH_3_ gives a notable increase of the dielectric constant, especially for NH_3_@MFM-300(Fe) (ε_*r*_ = 35.3 ± 0.3 at 10 kHz), which is comparable to MOFs with the highest dielectric constants (Table S3). PXRD analysis confirmed the stability of the regenerated MFM-300(M) after impedance measurements (Figure S2). The dielectric constant for bare MFM-300(Fe) shows little variation over the frequency range of 1 kHz to 1 MHz (Figure 1b, black curve, 5.64 at 1 kHz and 5.10 at 1 MHz). In contrast, the dielectric constant of NH_3_@MFM-300(Fe) shows a stronger frequency dependence, with the ε_*r*_ decreasing from 51.4 (1 kHz) to 12.4 (1 MHz) (Figure 1b, red curve). This suggests that the notable increase of the dielectric constant of NH_3_@MFM-300(Fe) is likely related to interfacial polarization due to electrical inhomogeneity within the MOF framework.^28^ This is further confirmed by the Z^*^ plots (Figure S4a), and exposure to 100% NH_3_ leads to a marked reduction in resistance. In addition, bare MFM-300(Fe) shows a small dielectric loss (tan δ = 0.006 at 10 kHz; Figure S4b), suggesting a static state of ions or dipoles over the entire structure. In contrast, the tan δ for NH_3_@MFM-300(Fe) increases markedly to 0.374 at 10 kHz due to energy loss from mobile NH_3_ guest molecules that are not bound directly to the MOF host.

**Figure 2:**
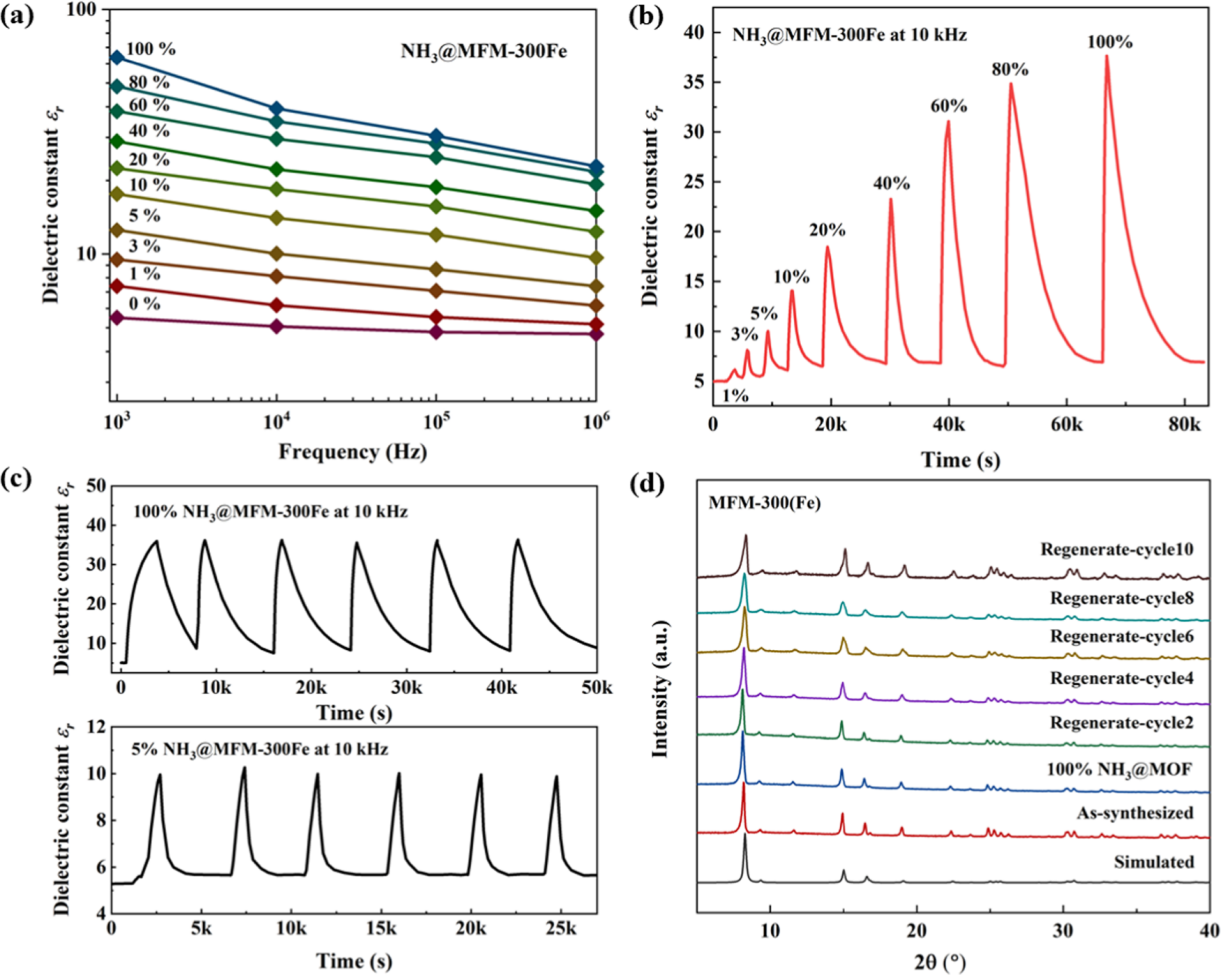
(a) Variation of the dielectric constant of MFM-300(Fe) as a function of the concentration of NH_3_ ranging from 0 to 100% at 25 °C and 1.0 bar in total. (b) Dynamic gas flow experiments upon loading various concentrations of NH_3_ into MFM-300(Fe) at 25 °C and 1.0 bar. (c) Cycling tests of the dielectric constant of MFM-300(Fe) under 100% NH_3_ and 5% NH_3_ at 10 kHz. (d) PXRD patterns of simulated and as-synthesized NH_3_@MFM-300(Fe) and of regenerated MFM-300(Fe); the loading and regeneration have been conducted for up to 10 cycles.

The excellent dielectric response of MFM-300(Fe) led us to evaluate its sensitivity and stability as a potential NH_3_ sensor. Figure 2a,b shows the corresponding values of ε_*r*_ with the same adsorption time of 1000 s as a function of frequency and NH_3_ concentration. The dielectric constant of MFM-300(Fe) increases gradually over the whole frequency range from 1 kHz to 1 MHz, demonstrating the adsorption of NH_3_. Taking the dielectric constant at 10 kHz as an example (Figure 2b), it is observed that 0% NH_3_@MOF (ε_*r*_ = 5.28) < 5% NH_3_@MOF (ε_*r*_ = 10.0) < 40% NH_3_@MOF (ε_*r*_ = 22.2) < 100% NH_3_@MOF (ε_*r*_ = 35.3). Additionally, MFM-300(Fe) also shows good sensitivity when exposed to 1% NH_3_ (10^4^ ppm), with a 20% increase of its dielectric constant to 6.18 (compared with the bare MOF of 5.12 at 10 kHz).

The cyclic measurements of the dielectric constant at both high (100%) and low (5%) concentrations of NH_3_ in MFM-300(Fe) were undertaken (Figure 2c). The dielectric constant is reproducible during the cyclic adsorption and desorption process, and is in good agreement with the reversible adsorption of NH_3_. The excellent stability of MFM-300(Fe) after cycling experiments is further confirmed by PXRD (Figure 2d). By contrast, the other analogues of MFM-300(M) (M = Al, Sc, Cr, Ga, In) exhibit smaller increases [up to 9.07 ± 0.26, compared with 35.3 ± 0.3 for MFM-300(Fe)] in their dielectric constants upon adsorption of NH_3_ (Figure 1a and Table S1). PXRD analysis confirms that both MFM-300(In) and MFM-300(Ga) have limited stability to NH_3_ over multiple cycles of sorption (Figure S7). The high stability and notable increase of the dielectric constant of MFM-300(Fe) upon NH_3_ adsorption demonstrate the potential of this MOF as a candidate for the NH_3_ sensor.

### Structural and Spectroscopic Studies

**Figure 3:**
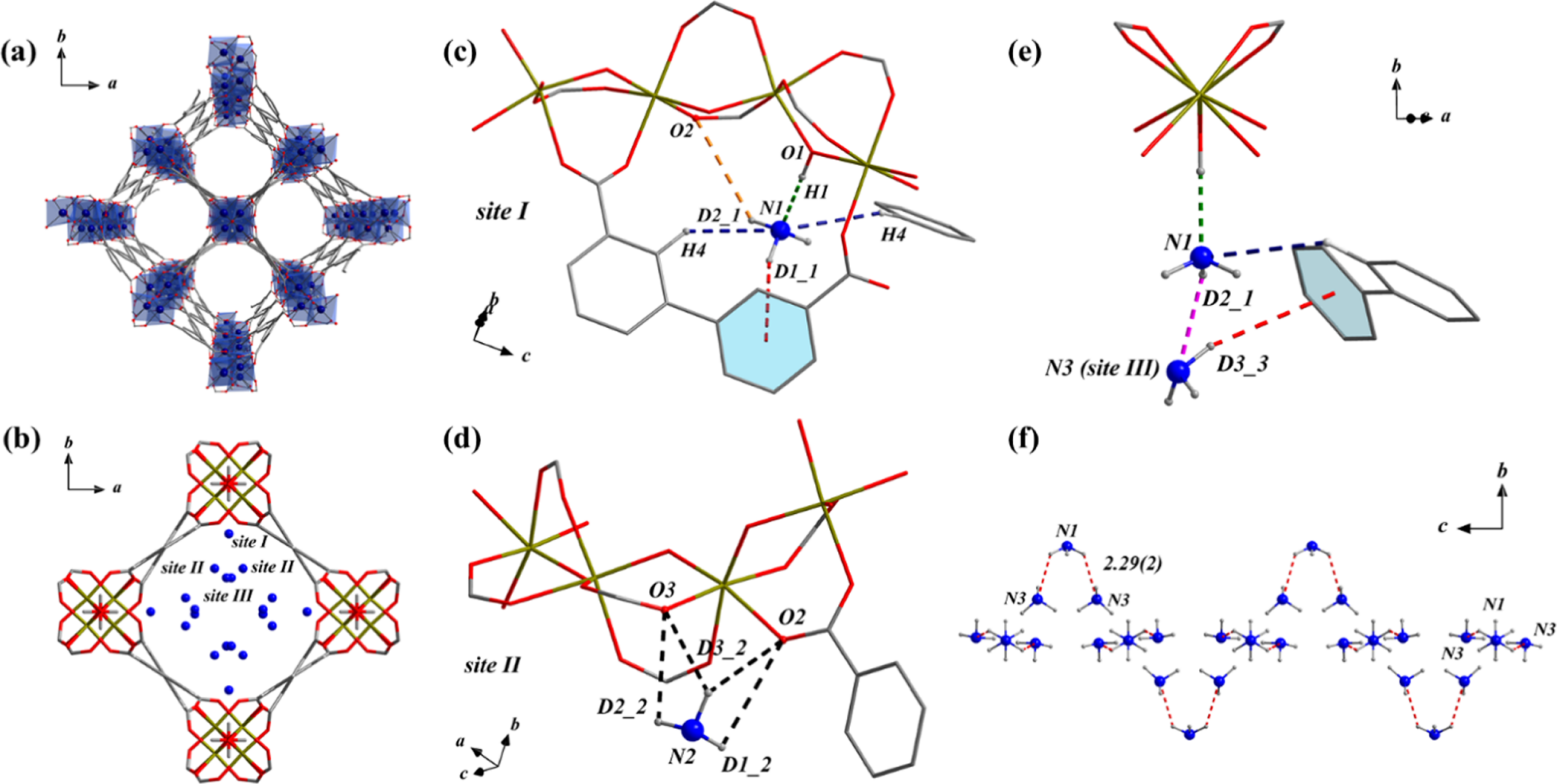
View of the crystal structure of (a) MFM-300(Fe) and (b) ND_3_-loaded MFM-300(Fe)·4.4ND_3_ along the *c* axis. (c–e) Views of the host–guest interactions between MFM-300(Fe) and adsorbed ND_3_ molecules. (f) View along the *a* axis showing a cooperative {ND_3_}_∞_ network running along the channel of MFM-300(Fe).

We sought to understand the origin of the *ca.* 7-fold increase of the dielectric constant of MFM-300(Fe) upon binding of NH_3_ by analyzing the host–guest interactions. Figure 3 shows three binding sites of adsorbed ND_3_ molecules in MFM-300(Fe)·4.4ND_3_ as determined by neutron powder diffraction (NPD) at 10 K (Table S4).^21^ Site I possesses full occupancy with four types of host–guest interactions. The hydrogen bond between the bridging hydroxyl group of MFM-300(Fe) and adsorbed ND_3_ molecules [O1_bridge_–H1···N1 = 1.90(3) Å] is the dominating interaction (Figure 3c), and two further hydrogen bonds [C4–H4_aromatic_···N1 = 3.14(1) Å and N1–D···O2_ligand_ = 3.17(1) Å] illustrate the interactions between ND_3_ molecules and the ligand. This is supplemented by an electrostatic interaction [N1–D···aromatic ring = 2.947(1) Å]. ND_3_ molecules at Site II [occupancy of 0.79(1)] are stabilized by four types of hydrogen bonds (Figure 3d). ND_3_^II^, as the hydrogen donor, interacts with two oxygen atoms (O2 and O3) from the carboxylate groups of the ligand [D1···O2 = 2.99(3) Å; D2···O3 = 2.75(3) Å; D3···O2 = 2.81(3) Å; D3···O3 = 2.61(3) Å]. ND_3_ molecules at Site III [occupancy of 0.44(1)] are stabilized by electrostatic interactions [N3–D···aromatic ring = 3.38(14) Å] (Figure 3e). Moreover, ND_3_^III^ forms intermolecular hydrogen bonds with ND_3_^II^ [N1–D···N3 = 2.29(2) Å]; this intermolecular interaction between ND_3_ molecules contributes to the formation of a cooperative {ND_3_}_∞_ network (Figure 3f).

**Figure 4:**
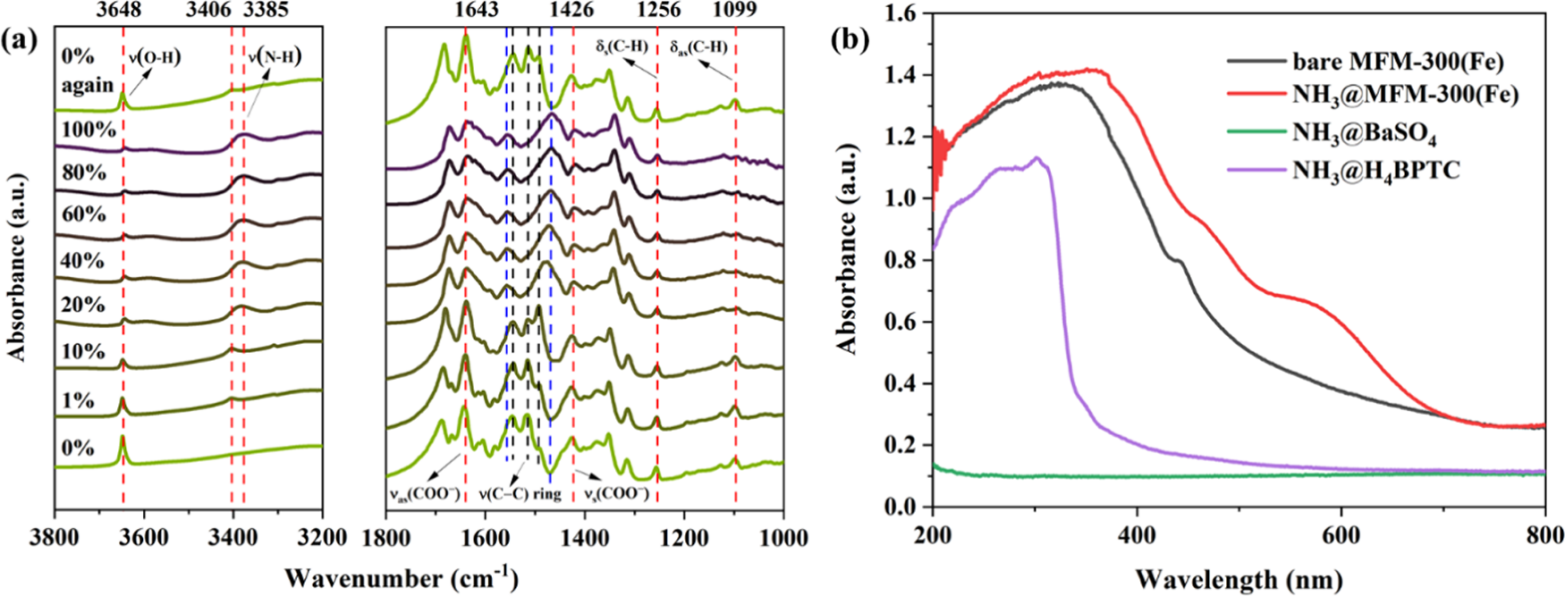
(a) *In situ* synchrotron FTIR spectra for MFM-300(Fe) as a function of the concentration of NH_3_ (diluted in dry N_2_) and after regeneration under a dry N_2_ flow at 10 mL min^–1^ at 150 °C for 2 h. (b) Solid-state UV/vis diffuse reflectance spectra of NH_3_@BaSO_4_, NH_3_@H_4_BPTC, MFM-300(Fe), and NH_3_@MFM-300(Fe).

Host–guest interactions in this system were studied further using *in situ* synchrotron Fourier transform infrared (FTIR) microspectroscopy as a function of NH_3_ loading (Figure 4a and Table S5).^21^ The binding of NH_3_ molecules in MFM-300(Fe) is evidenced by the emergence of a band at 3406 cm^–1^ (N–H stretching) upon adsorption, which exhibits a red shift to 3385 cm^–1^ when the NH_3_ loading is increased to 20%. This suggests that the vibrations of adsorbed NH_3_ molecules are further restricted by the intermolecular interactions within the {ND_3_}_∞_ network (Figure 3f). Moreover, the N–H band broadens at high loadings, indicative of a more complex binding environment. In contrast, the characteristic stretching mode of the bridging hydroxyl group of MFM-300(Fe) is observed at 3648 cm^–1^. This band decreases in intensity and shows a red shift with the increasing NH_3_ loading due to the host–guest interaction (O1_bridge_–H1···ND_3_^I^) (Figure 3c). Similarly, bands for the asymmetric and symmetric stretching vibrations of the COO^–^ group at 1643 and 1426 cm^–1^, respectively,^29,30^ show red shifts as well as a lower intensity with increased NH_3_ loading, consistent with the interactions of ND_3_···O_ligand_ (Figures 3c,d and 4a). Also, as the percentage of NH_3_ is increased from 10 to 20%, three bands at 1548, 1516, and 1494 cm^–1^ assigned to the C–C stretching in aromatic rings^31,32^ merge into two bands at 1558 and 1469 cm^–1^ (Figure 4a). This indicates a change of the conjugated structure of aromatic rings, consistent with the elongation of C–C bonds in the biphenyl linker from ∼1.386 Å in bare MFM-300(Fe) to ∼1.419 Å in NH_3_-loaded MFM-300(Fe) (Table 1). Interestingly, this phenomenon is not observed in NH_3_-loaded MFM-300(Al) in which the C–C bond distances show little change (Table 1), thus confirming that choice of M in this series can have a significant effect on properties. Due to the interaction of H_aromatic_···ND_3_ (Figure 3c,e), the two bands of C–H deformational modes observed at 1256 and 1099 cm^–1^ in the FTIR spectrum also decrease in intensity upon NH_3_ loading into MFM-300(Fe) (Figure 4a).^33,34^

**Table 1:** Comparison of Bond Distances in Bare and ND_3_-Loaded MFM-300(Fe) and MFM-300(Al)

	bond distance	MFM-300(Fe)^a^	MFM-300(Fe)·4.4ND_3_^a^	MFM-300(Al)^a^	MFM-300(Al)·2.4ND_3_^a^
aromatic ring	C2–C3	1.386(2)	1.419(4)	1.417(6)	1.417(6)
	C2–C4	1.386(2)	1.418(4)	1.418(8)	1.418(9)
	C4–C5	1.386(1)	1.419(3)	1.418(4)	1.417(4)
M–O	M–O1	1.977(2)	1.907(5)	1.858(8)	1.879(11)
	M–O2	2.034(4)	2.124(11)	2.002(16)	2.043(18)
	M–O3	2.003(4)	2.046(10)	1.893(17)	1.934(20)

a Crystal structures were all determined by NPD at 10 K.

Solid-state UV/vis spectra of bare and NH_3_-loaded MFM-300 samples were recorded to reveal the electronic structure of MFM-300 upon NH_3_ adsorption (Figure 4b). The solid-state UV/vis spectra of the Al, Ga, In, Sc, and Cr analogues display little difference upon adsorption of NH_3_ (Figure S6). In contrast, a distinct new band at 600 nm and a red shift of the ligand-to-metal charge transfer (LMCT) band from 370 to 400 nm were observed in the spectrum of MFM-300(Fe) upon adsorption of NH_3_ (Figure 4b). This is an indication of the redistribution of electron densities in the porous framework induced by NH_3_ adsorption. This is consistent with the structural analysis based on the NPD results,^21^ in which two electrostatic interactions [ND_3_^I^···aromatic ring = 2.947(1) Å and ND_3_^III^···aromatic ring = 3.38(14) Å] elongate the C–C bonds in the biphenyl linker from ∼1.386 Å in the bare MOF to ∼1.419 Å in the NH_3_-loaded MOF (Table 1). The weakening of the C–C bonds suggests the involvement of delocalized π-electrons in LMCT, thus inducing the red shift of the LMCT band. In addition, the elongated bond distances of Fe–O2 and Fe–O3 in ND_3_@MFM-300(Fe) [2.124(11) and 2.046(10) Å, respectively], compared with 2.034(4) Å and 2.003(4) Å in MFM-300(Fe), reflect host–guest interactions of ND_3_^II^···O_ligand_, in good agreement with the enhanced electron delocalization. This leads to stronger structural polarization within the MOF. In contrast, Al, Ga, In, and Sc analogues show little change in the dielectric constant upon adsorption of NH_3_ since the cations M(III) have an inert gas configuration (ns^2^np^6^), while the charge transfer is observed for cations with partly occupied *d* orbitals. Yet, though Cr(III) cations have a *d*^3^-electron configuration, the dielectric constant of NH_3_@MFM-300(Cr) is only 6.54 ± 0.27, only moderately higher than that of MFM-300(Cr) (ε_*r*_ = 3.64 ± 0.08). Cr(III) has a larger atomic radius (1.22 Å) than Fe(III) (1.16 Å), resulting in longer Cr–O bonds than Fe–O bonds (1.85 Å and 1.79 Å, respectively) based upon the calculated single-bond covalent radii,^35^ affording the weaker electronic delocalization. The solid-state UV/vis spectra of NH_3_@MFM-300(Cr) show little difference compared with MFM-300(Cr), indicating no obvious increase in electron delocalization upon adsorption of NH_3_.

## Conclusions

In summary, the dielectric properties of the complexes MFM-300(M) (M = Al, Sc, Cr, Fe, Ga, In) upon adsorption of Ar and NH_3_ have been investigated. Of this series, MFM-300(Fe) shows high stability and sensitivity to NH_3_ with the highest dielectric constant of 35.3 ± 0.3 at 10 kHz upon adsorption of NH_3_. The enhanced dielectric constant of MFM-300(Fe) on binding to NH_3_ arises from structural polarization induced by host–guest hydrogen-bonding interactions as determined by neutron powder diffraction studies. This phenomenon is linked to electron delocalization within the Fe-linker, supported by the observation in the solid-state UV/vis spectra of changes in ligand-to-metal charge-transfer transitions. This provides further support for the mechanism of structural polarization, which will inspire the design of new dielectric MOF-based sensors based around optimized host–guest interactions.

## Supporting Information Available

Bulk pellets of MFM-300(M) were placed into an electrochemical gas cell equipped with electrodes (Figure S1) (PDF)
Porous metal–organic framework (MOF) (CIF)
Porous metal–organic framework (MOF) (CIF)

## Supplementary Material

ic3c02110_si_001.pdf

ic3c02110_si_002.cif

ic3c02110_si_003.cif
