# Supplementary material for: Impact of Host–Guest Interactions on the Dielectric Properties of MFM-300 Materials
Source: Inorg Chem. 2023 Oct 9;62(42):17157–62. doi: 10.1021/acs.inorgchem.3c02110 (PMC10598873; doi:10.1021/acs.inorgchem.3c02110)
Supplement: Supplementary file 1 — ic3c02110_si_001.pdf [file ic3c02110_si_001.pdf]

Supporting Information

The impact of host-guest interactions on the dielectric properties of MFM-300 materials

Xi Chen[1], Sergei Sapchenko[1], Wanpeng Lu[1], Ming Li[2], Meng He[1], Yinlin Chen[1], Mark D. Frogley[3], Ivan da Silva[4], Sihai Yang[1]* and Martin Schröder[1]*

1. Department of Chemistry, University of Manchester, Manchester, M13 9PL, U.K.

Sihai.Yang@manchester.ac.uk; M.Schroder@manchester.ac.uk

2. Faculty of Engineering, University of Nottingham, Nottingham NG7 2RD, U.K.

3. Diamond Light Source, Harwell Science Campus, Oxfordshire OX11 0DE, U.K.

4. ISIS Facility, Science and Technology Facilities Council (STFC), Rutherford Appleton Laboratory, Didcot OX11 0QX, U.K.

Experimental section

Synthesis

Starting materials were purchased from Acros Organics and Sigma-Aldrich and used without further purification. The ligand H_4BPTC was synthesised based on our pervious report.[1] The materials MFM-300(M), (M = Al, Cr, Fe, Ga, In, Sc) were synthesized through solvothermal methods.[2-6]

MFM-300(Al) was synthesised according to our previous method.[2] H_4BPTC (0.06 g, 0.182 mmol), $Al(NO_3)_3{\cdot}9H_2O$ (0.34 g, 0.906 mmol), piperazine (0.10 g, 1.26 mmol), deionised water (10 mL) and HNO_3 (2.8M, 2.0 mL) were mixed to form a white slurry, and heated in an autoclave at 210 °C for 3 days. The product was collected by filtration, washed with water for several times and dried in air.

MFM-300(Fe) was synthesised according to our previous work.[3] H_4BPTC (0.098 g, 0.30 mmol), $FeCl_3$ (0.192 g, 1.18 mmol), dimethylformamide (DMF, 8 mL) and HCl (36.5 wt%, 0.3 mL) were mixed to form a yellow slurry, and heated in an autoclave at 120 °C for 3 days. The product was collected by filtration, washed with water, DMF and acetone several times and dried in air.

MFM-300(Cr) was synthesised according to our previous work.[4] H_4BPTC (0.70 g, 2.1 mmol), $CrCl_3{\cdot}6H_2O$ (2.83 g, 10.6 mmol), deionised water (100 mL) and HCl (1 wt%, 15 mL) were mixed, and heated in an autoclave at 210 °C for 3 days. The product was collected by filtration, washed with water, DMF and acetone several times and dried in air.

MFM-300(Sc) was synthesised according to our previous work.[5] H_4BPTC (0.1 g, 0.3 mmol), Scandium triflate (0.3 g, 0.61 mmol), deionised water (15 mL) and HCl (36.5 wt%, 3 mL) were mixed, and heated in an autoclave at 80 °C for overnight. The product was separated by filtration, washed with water, DMF and acetone several times and dried in air.

MFM-300(In) was synthesised according to our previous work.[3] H_4BPTC (0.33 g, 1.0 mmol), $In(NO_3)_3{\cdot}5H_2O$ (0.585 g, 1.65 mmol), acetonitrile (MeCN, 10 mL), DMF (20 mL) and HNO_3 (70%, 1.0 mL) were mixed, and heated in an autoclave at 90 °C for 3 days. The product was separated by filtration, washed with water, DMF and acetone several times and dried in air.

MFM-300(Ga) was synthesised according to our previous work.[6] H_4BPTC (0.218 g, 0.67 mmol), $Ga(NO_3)_3{\cdot}9H_2O$ (0.378 g, 1.48 mmol), DMF (20 mL), tetrahydrofuran (THF, 50 mL), deionised water (10 mL) and HCl (37%, 2 mL) were mixed, and heated in an autoclave at 100 °C for 3 days. The product was separated by filtration, washed with water, DMF and acetone several times and dried in air.

Characterisation

X-ray powder diffraction patterns were collected on a Phillips X'pert Pro MPD powder diffractometer (40 kV and 30 mA) using Cu-$K\alpha$ radiation (λ = 1.5406 Å at room temperature in a 2θ range of 3–50° with a scan speed of 0.4° min^{-1}. Structural determination of the binding positions of ND_3 within MFM-300(Fe) was conducted on WISH, a long-wavelength powder and single crystal neutron diffractometer at the ISIS neutron and muon facility at Rutherford Appleton Laboratory (UK). *In situ* gas-loaded single-crystal synchrotron FTIR microspectroscopy was carried out at the Multimode InfraRed Imaging and Microspectroscopy (MIRIAM) beamline at the Diamond Light Source, Harwell Science Campus (UK). The solid UV–Vis diffuse reflectance spectroscopic data were recorded on a UV–Vis spectrophotometer (Shimadzu, UV 2600) equipped with an integrating sphere using $BaSO_4$ as reference.

Dielectric constant measurements

Dielectric constant measurements were performed on the Solartron SI1260 Impedance analyser over a frequency range of 1 Hz to 1 MHz at the amplitude of 100 mV under the DC rest voltage of 0 mV at ambient condition. The impedance analyser was connected by an electrochemical gas cell equipped with platinum current collectors (Figure. S1). All samples were pressed into the pellets ($d \approx 0.1$ cm, $\phi = 0.8$ cm) after grinding into fine powder under 3 tons pressure for 3 min, and a Ag/Au paste was coated on the top/bottom face of each pellet. The pellet was transferred to the gas cell and activated under vacuum (1.0×10^{-2} mbar) at different temperature (120 -170 °C) for 12 h before dosing different gases. The dielectric constants in Figure 1a were measured through static gas dosing experiments at 0.5 bar under room temperature. The gas cell was placed under vacuum and then loaded with different gases by using a dosing rig. The dielectric constant was recorded when the NH_3 adsorption had reached saturation to give a constant dielectric constant (Figure S4). The results in Figure 2a-b were tested through the dynamic gas flow experiment at 1 bar at room temperature. Research-grade Argon (Ar) and ammonia (NH_3) were purchased from BOC and used as received. Ar was used as a carrier gas to dilute NH_3 to the desired concentration. The gas flow rate was controlled by a mass flow meter (MFM). The dielectric constant (ε_r) was calculated from the electrical capacitance (C) according to equation (1).

$$C = \varepsilon_r \cdot \varepsilon_0 \cdot \frac{A}{d} \tag{1}$$

where C is capacitance, ε_r is the relative dielectric constant (the real dielectric constant of the sample), ε_0 is the dielectric constant of vacuum ($\varepsilon_0 = 8.85\times10^{-12}$ F/m), A is the cross-sectional area of the sample pellet, and d is the thickness of the pellet.

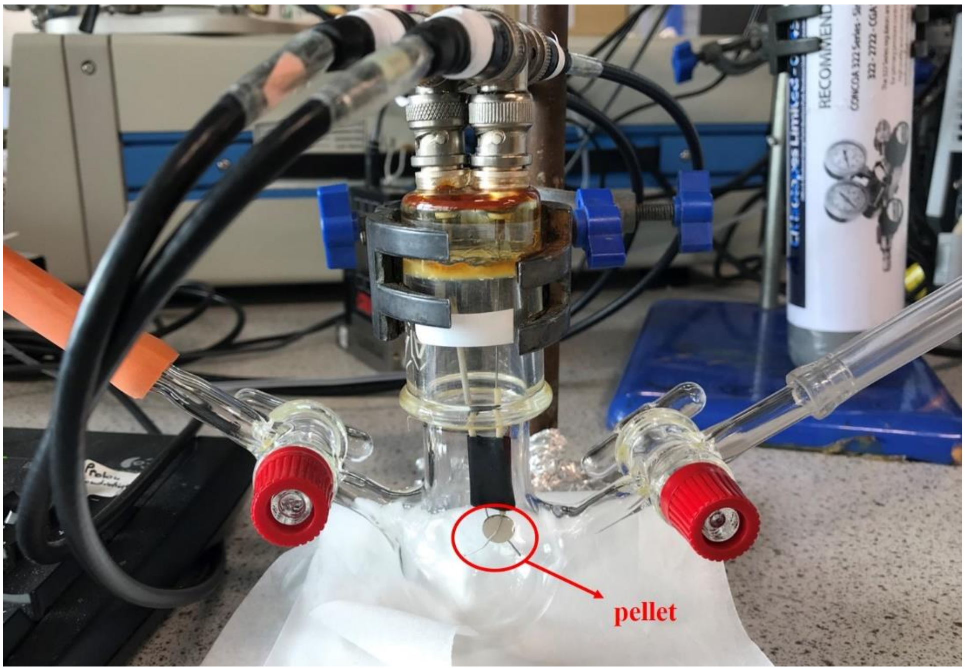

Figure S1. View of the electrochemical gas cell used in this work.

PXRD analysis of MFM-300(M) (M = Al, Cr, Fe, Ga, In, Sc)

All PXRD patterns of as-synthesised MFM-300 are in good agreement with the simulated patterns, thus confirming the phase purity. After the first cycle of impedance measurement upon NH_3 adsorption, the Ag/Au paste on the MOF pellet was removed, and the pellet ground into fine powder. The PXRD patterns of the regenerated MOFs after NH_3 adsorption of the first cycle match well with those of the as-synthesised samples, confirming the NH_3 stability of MFM-300(M) in the impedance measurement.

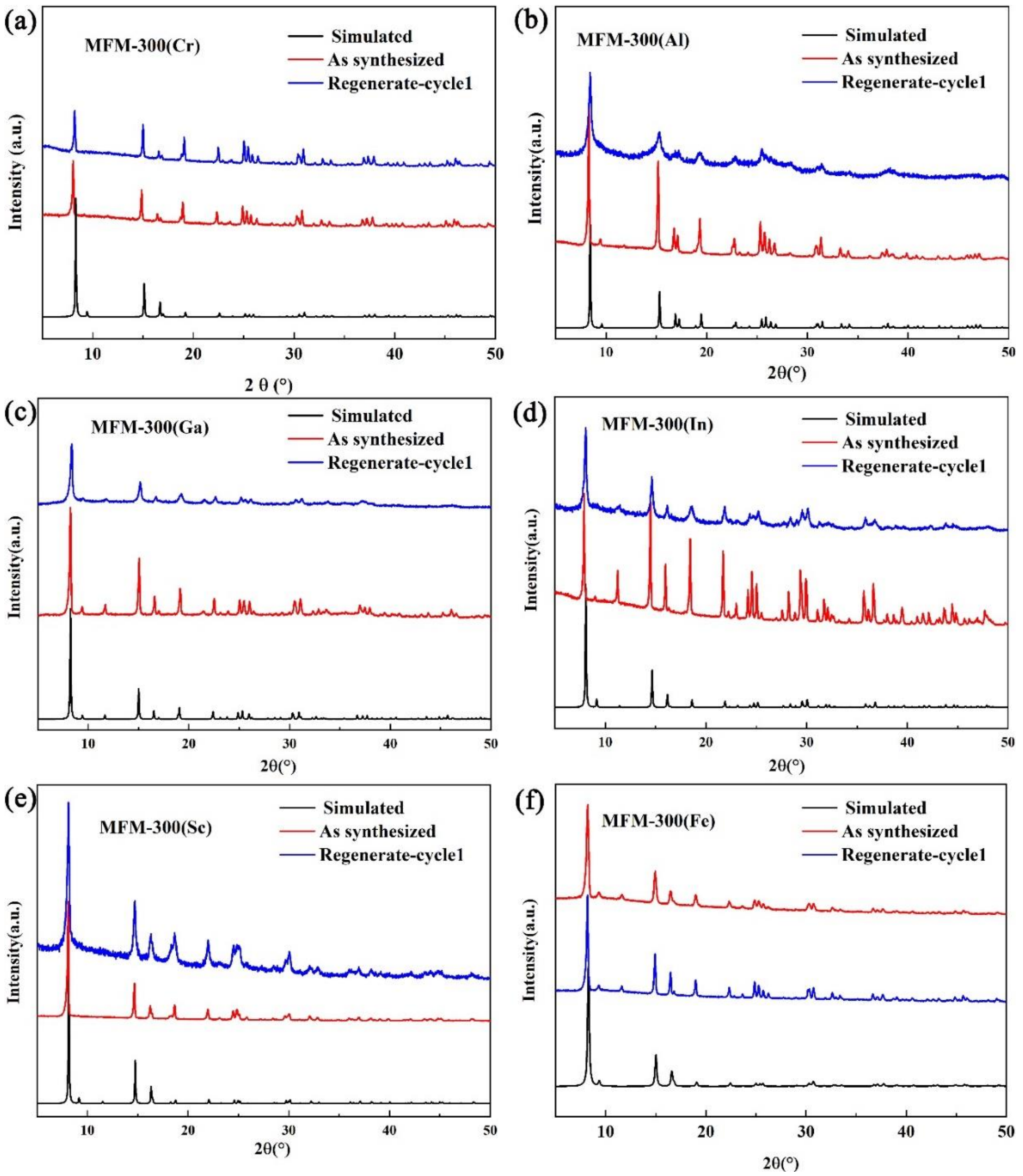

Figure S2. PXRD patterns of simulated (black), as-synthesised (red) and after impedance measurements after 1st cycle of NH_3 adsorption (blue).

Dielectric property of MFM-300 (M) (M = Al, Sc, Cr, Fe, Ga, In)

The dielectric constants of MFM-300(M) are summarised in Table S1. The standard errors were calculated based on three repeated measurements using different batches of the bulk pellets of MFM-300(M) samples.

Table S1. Summary of BET surface areas, NH_3 uptake capacities[4,7-9] and dielectric constants measured at 10 kHz for MFM-300(M) under vacuum and under loadings of Ar and NH_3 at the pressure of 0.5 bar.

**MFM-300(M)**	**Al**	**Sc**	**Cr**	**Fe**	**Ga**	**In**
BET surface area (m^2/g) based on previous reports	1325	1390	1045	1192	1064	1030
NH_3 uptake capacity at 273K and 1 bar (mmol/g) based on previous reports	15.7	19.5	14.0	16.1	unstable	unstable
Dielectric constant under vacuum	3.81±0.16	5.19±0.05	3.64±0.08	5.12±0.25	4.67±0.08	5.08±0.08
Dielectric constant under Ar	3.87±0.14	5.26±0.01	3.66±0.10	5.28±0.10	4.74±0.12	5.11±0.13
Dielectric constant Under loading NH_3 at 0.5 bar	6.76±0.17	6.69±0.10	6.54±0.27	35.3±0.30	9.07±0.26	5.60±0.27

Table S2. Summary of dielectric constants of three different samples of NH_3-loaded MFM-300(Fe) at 10 kHz.

	d **(cm)**	**cycle**	**condition**	ε_r
Sample1	0.12	1	vacuum	5.14
			100% NH_3	35.6
				35.5
				35.2
		2	vacuum	5.28
			100% NH_3	34.5
				34.4
				34.2
Sample2	0.095	1	vacuum	5.18
			100% NH_3	37.0
		2		37.2
		3		37.3
		4		36.8
Sample3	0.11	1	vacuum	5.21
			100% NH_3	35.8
				34.8
				34.2
		2	100% NH_3	34.1
				34.3
				33.7
MFM-300(Fe)			100% NH_3	35.3±0.30

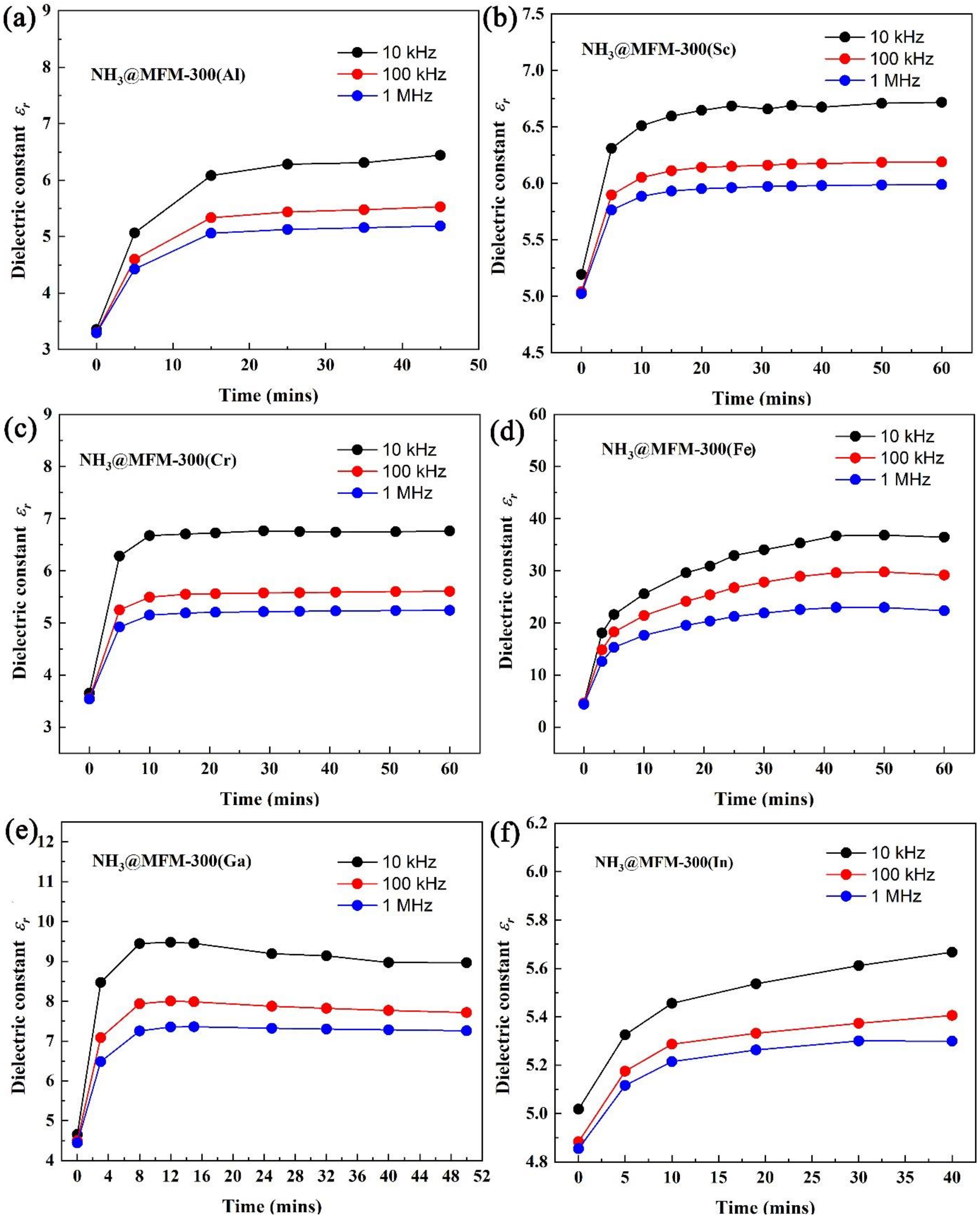

Figure S3. Dielectric constants for NH_3-loading of MFM-300 (M) (M= Al, Cr, Fe, Ga, In, Sc).

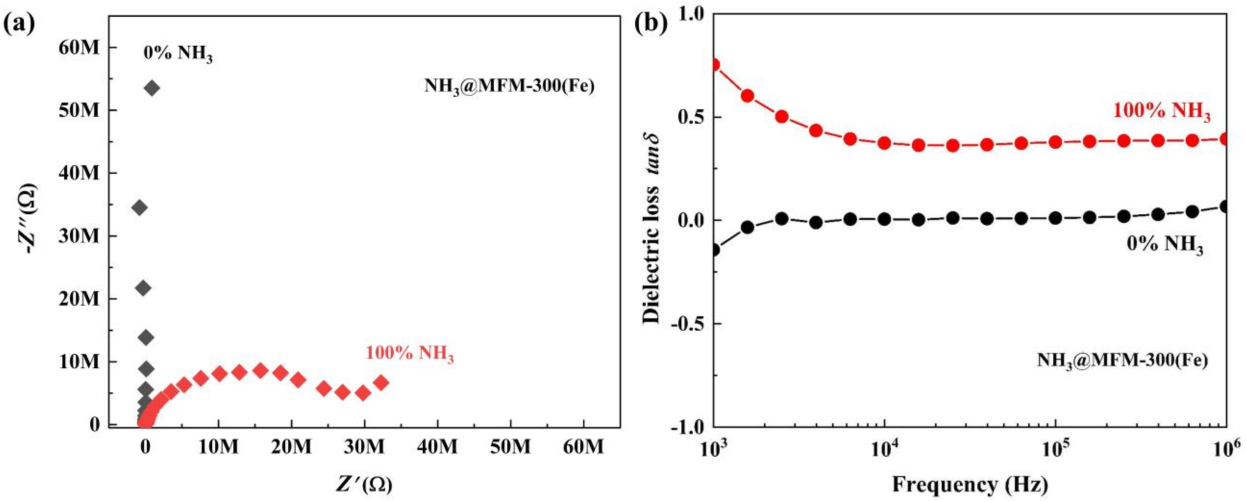

Figure S4. (a) Z^* plots of bare MFM-300(Fe) and NH_3@MFM-300(Fe). (b) Dielectric loss of MFM-300(Fe) as a function of frequency before and after NH_3 adsorption.

Dielectric property of reported MOFs

Table S3. Comparison of dielectric constants for related systems from the literature

MOFs	Sample preparation	Guests	T (°C)	Frequency	ε_r
MIL-100(Fe)[10]	Pellet, 3t	bare	20-30	10 kHz<br>0.1 MHz<br>1MHz	5.5<br>5.3<br>5.1
H-KUST1[11]	single crystal	N_2	25	1MHz	2.75±0.25
		I_2			2.71±0.22
		EtOH			7.45±0.10
		MeOH			10.13±0.10
		H_2O			55.73±1.87
H-KUST1[12]	pellet, 3t	bare	20-30	10 kHz<br>0.1 MHz<br>1MHz	4.1<br>3.9<br>3.7
H-KUST1[13]	pellet, 0.2 GPa	H_2O and MeOH	25	1 MHz	5.16
		H_2O (air)			3.02
		Empty			1.72
$[Sm_2(bhc)(H_2O)_6]_n$[14]	Pellet	Pristine	37	10 kHz	45.1<br>44.5<br>42.5
$\{[Zn_2(Ltrp)_2(bpe)_2(H_2O)_2]\cdot 2H_2O \cdot 2NO_3\}_n$[15]	Pellet	Pristine	25	1MHz	2.53
		H_2PO_4-exchanged			3.50
Sr(TDA)(DMF)[15]	Pellet	Pristine	50	10 kHz	14.0
$[Sr(BDC)(DMA)(H_2O)]$[15]	Pellet	Pristine	20	10 kHz	15.3
[Sr(ABDC)(DMF)][15]					34.0
[Ca(ABDC)(DMF)][16]					19.3
$[Ca(ABDC)(DMA)_{2/3}]$[16]					12.5
ZIF-8[17]	film	Pristine	25	0.1 MHz	2.33±0.05
$[Zn(CEIC)_2(H_2O)]\cdot 2DMF$[18]	Pellet	Pristine	30	10 kHz	3.90
$[Zn_2(Hbbim)_2(bbim)]_n$[19]	Pellet	bare	25	1MHz	3.05
$\{Zn(TBTC)[H_2N(CH_3)_2]\}$ .2DMF EtOH[20]	bulk	Pristine	30	1MHz	5.9
	Film				19.5
MFM-300(Fe)	Pellet	bare	25	10 kHz	5.12±0.25
		5% NH_3		10 kHz	10.0±0.06
		100% NH_3		10 kHz	35.3±0.3

HKUST-1, $Cu_3(BTC)_2$, H_3BTC = benzene-1,3,5-tricarboxylic acid; H_6bhc = benzenehexacarboxylic acid; LHtrp = L-tryptophan, bpe = 1,2-bis(4-pyridyl)ethylene); H_2TDA = 2,5-dicarboxylic acid; H_2BDC = 1,4-benzenedicarboxylic acid; H_2ABDC = 2-aminobenzenedicarboxylic acid; DMF = N, N'-dimethyl formamide; DMA = N,N'-dimethyl acetamide; HCEIC = 4-carboxy-2-ethyl-1H-imidazole-5-carboxylic acid; H_2bbim = bis-benzimidazole

Crystal structure information of ND_3@MFM-300(Fe)

Table S4 Host-guest interactions of three binding sites of ND_3 in MFM-300(Fe)

Site	D	H	A	d(D−H)/Å	d(H−A)/Å	d(D−A)/Å	D−H−A/°	Figure 3c-e
I	O1 (bridge)	H1	N1 (site I)	0.90(3)	1.90(3)	2.80(1)	179.9(1)	Green dashed line
	C4 (aromatic)	H4	N1 (site I)	1.05(1)	3.14(1)	4.02(1)	142.5(4)	Blue dashed line
	N1 (site I)	D1_1	Aromatic ring	1.01(1)	2.947(1)	3.92(1)	162.05(1)	Red dashed line
	N1 (site I)	D2_1	O2 (ligand)	1.01(1)	3.17(1)	3.92(1)	131.8(1)	Yellow dashed line
II	N2 (site II)	D1_2	O2 (ligand)	1.01(2)	2.99(3)	3.36(2)	102.9(16)	Black dashed line
	N2 (site II)	D2_2	O3 (ligand)	1.02(3)	2.75(3)	3.13(1)	102.5(19)	
	N2 (site II)	D3_2	O2 (ligand)	1.01(3)	2.81(3)	3.36(2)	114.4(17)	
	N2 (site II)	D3_2	O3 (ligand)	1.01(3)	2.61(3)	3.13(1)	111.7(17)	
III	N1 (site I)	D2_1	N3 (site III)	1.01(1)	2.29(2)	2.89(2)	116.6(4)	Pink dashed line
	N3 (site III)	D3_3	Aromatic ring	1.01(12)	3.38(14)	4.18(1)	137.1(90)	Red dashed line

DRIFTS spectra of MFM-300(M), (M = Al, Cr, Fe, Ga, In, Sc)

IR spectroscopic data for MFM-300(M) were collected on a Diffuse Reflectance Infrared Fourier Transform (DRIFTS) instrument over the range 400-4000 cm^{-1} using dry N_2 with a total flow rate of 40 mL/min. The ν(OH) stretching mode of the M-O(H)-M moiety in MFM-300(M) (M = Al, Ga, In, Sc) appears at 3658-3685 cm^{-1}, higher than that of MFM-300(Fe) and MFM-300(Cr) (3645 and 3640 cm^{-1}, respectively), suggesting that the –OH group in MFM-300(Fe) and MFM-300(Cr) are more acidic and accessible to guest species (Figure. S5).

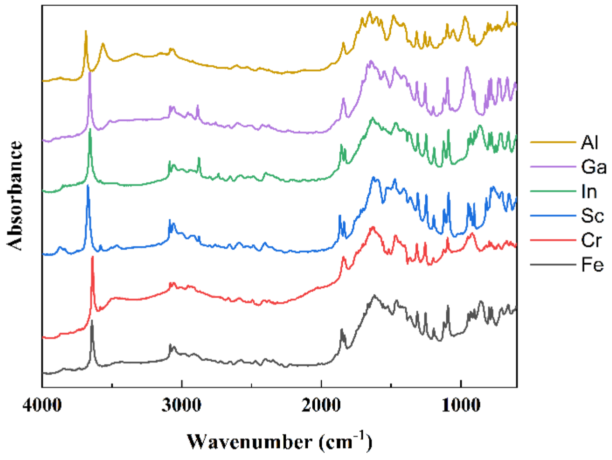

Figure S5. DRIFTS spectra of bare MFM-300(M) (M= Al, Cr, Fe, Ga, In, Sc) at 4000-600 cm^{-1} under dry N_2.

Table S5 Infrared spectral data (in cm^{-1}) for NH_3-loaded MFM-300(Fe) and proposed assignments.

Assignment	Bands (cm^{-1}) (0% loadings)	Bands (cm^{-1}) (20% loadings)	Red shift (cm^{-1})
ν(O-H)	3648	3644	-4
ν(N-H)	3406	3385	-21
ν_{as}(COO^-)	1643	1637	-6
ν(C-C) ring	1548, 1516, 1494	1558, 1469	—
ν_s(COO^-)	1426	1422	-4
ν(C-C) ring	1315	1311	-4
δ_s(C-H)	1256	1254	-2
δ_{as}(C-H)	1099	1093	-6

Solid-state UV/Vis spectra for MFM-300 (M) (M = Al, Cr, Ga, In, Sc)

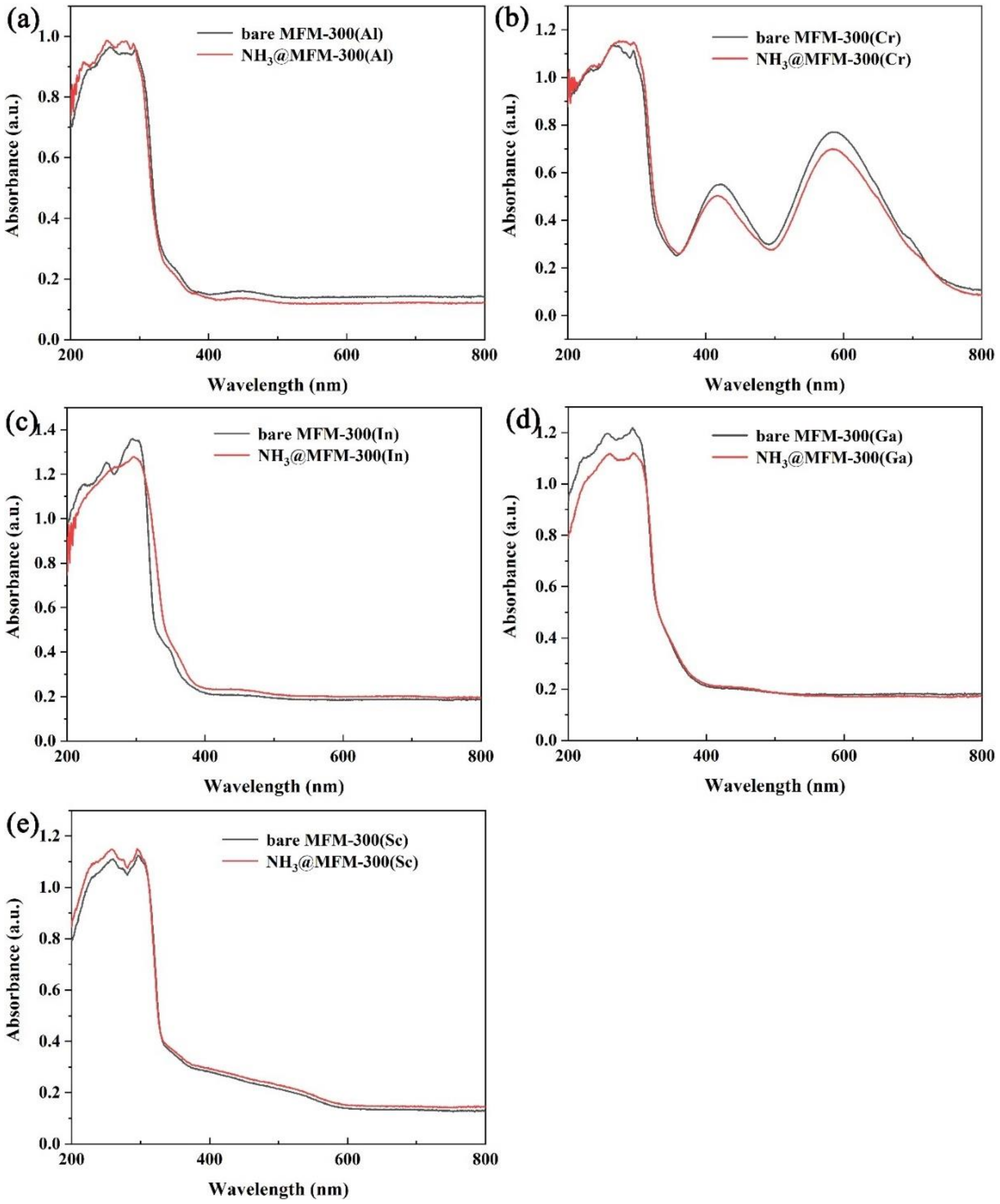

Figure S6. Solid-state UV/Vis diffuse reflectance spectra of bare and NH_3-loaded MFM-300(M).

Long term stability of MFM-300 (M) on exposure to NH_3

MFM-300(Al), MFM-300(Sc) and MFM-300(Cr) show good stability to NH_3 and retain good crystallinity after five cycles of NH_3 dosing experiments. However, PXRD patterns of regenerated MFM-300(M) (M = In, Ga) indicate that they are both unstable to NH_3 upon multiple cycling experiments.

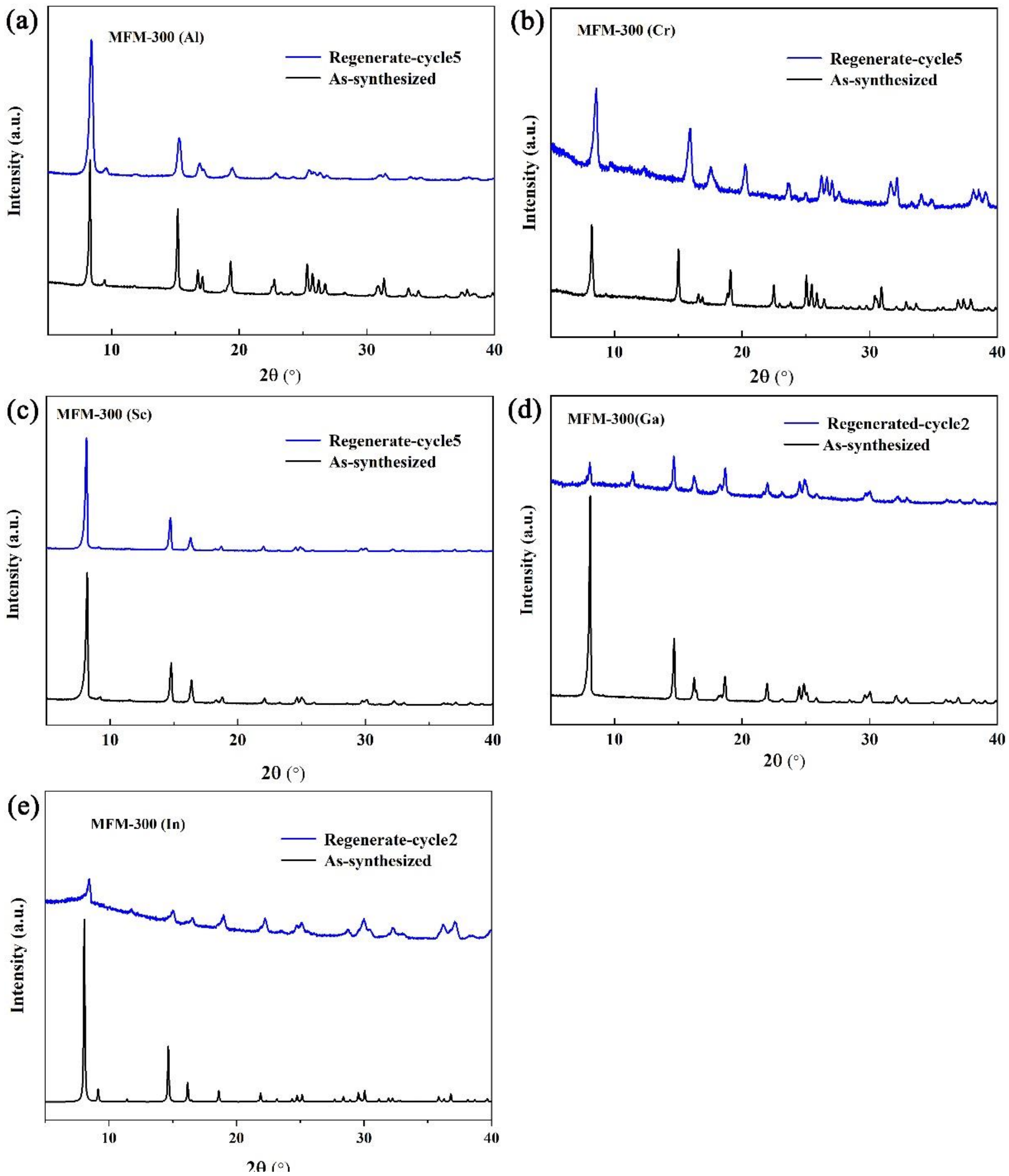

Figure S7. PXRD patterns for NH_3-loaded MFM-300(M) and regenerated MFM-300(M).

References

(1) Lin, X.; Telepeni, I.; Blake, A. J.; Dailly, A.; Brown, C. M.; Simmons, J. M.; Zoppi, M.; Walker, G. S.; Thomas, K. M.; Mays, T. J.; Hubberstey, P.; Champness, N. R.; Schröder, M. High Capacity Hydrogen Adsorption in Cu(II) Tetracarboxylate Framework Materials: The Role of Pore Size, Ligand Functionalization, and Exposed Metal Sites. *J. Am. Chem. Soc.* **2009**, *131*, 2159–2171.

(2) Yang, S.; Sun. J.; Ramirez-Cuesta, A. J.; Callear, S. K.; David W. I. F.; Anderson, D. P.; Newby, R.; Blake, A. J.; Parker, J. E., Tang, C. C.; Schröder, M. Selectivity and Direct Visualization of Carbon Dioxide and Sulfur Dioxide in a Decorated Porous Host. *Nat. Chem.* **2012**, *4*, 887-894.

(3) Zhang, X.; da Silva, I.; Godfrey, H. G. W.; Callear, S. K.; Sapchenko, S. A.; Cheng, Y.; Vitórica-Yrezábal, I.; Frogley, M. D.; Cinque, G.; Tang, C. C.; Giacobbe, C.; Dejoie, C.; Rudić, S.; Ramirez-Cuesta, A. J.; Denecke, M. A.; Yang, S.; Schröder, M. Confinement of Iodine Molecules into Triple-Helical Chains within Robust Metal–Organic Frameworks. *J. Am. Chem. Soc.* **2017**, *139*, 16289–16296.

(4) Han, X.; Lu, W.; Chen, Y.; da Silva, I.; Li, J.; Lin, L.; Li, W.; Sheveleva, A. M.; Godfrey, H. G. W.; Lu, Z.; Tuna, F.; McInnes, E. J. L.; Cheng, Y.; Daemen, L. L.; McPherson, L. J. M.; Teat, S. J.; Frogley, M. D.; Rudić, S.; Manuel, P.; Ramirez-Cuesta, A. J.; Yang, S.; Schröder, M. High Ammonia Adsorption in MFM-300 Materials: Dynamics and Charge Transfer in Host–Guest Binding. *J. Am. Chem. Soc.* **2021**, *143*, 3153–3161.

(5) Ibarra, I. A.; Yang, S.; Lin, X.; Blake, A. J.; Rizkallah, P. J.; Nowell, H.; Allan, D. R.; Champness, N. R.; Hubberstey, P.; Schröder, M. Highly Porous and Robust Scandium-Based Metal–Organic Frameworks for Hydrogen Storage. *Chem. Commun.* **2011**, *47*, 8304.

(6) Krap, C. P.; Newby, R.; Dhakshinamoorthy, A.; García, H.; Cebula, I.; Easun, T. L.; Savage, M.; Eyley, J. E.; Gao, S.; Blake, A. J.; Lewis, W.; Beton, P. H.; Warren, M. R.; Allan, D. R.; Frogley, M. D.; Tang, C. C.; Cinque, G.; Yang, S.; Schröder, M. Enhancement of CO_2 Adsorption and Catalytic Properties by Fe-Doping of [$Ga_2(OH)_2(L)$] (H_4L = Biphenyl-3,3′,5,5′-Tetracarboxylic Acid), MFM-300(Ga). *Inorg. Chem.* **2016**, *55*, 1076–1088.

(7) Guo, L.; Han, X.; Ma, Y.; Li, J.; Lu, W.; Li, W.; Lee, D.; da Silva, I.; Cheng, Y.; Rudić, S.; Manuel, P.; Frogley, M. D.; Ramirez-Cuesta, A. J.; Schröder, M.; Yang, S. High Capacity Ammonia Adsorption in a Robust Metal-Organic Framework Mediated by Reversible Host-Guest Interactions. *Chem. Commun.* **2022**, *58*, 5753–5756.

(8) Savage, M.; Carter, J. H.; Han, X.; da Silva, I.; Manuel, P.; Rudić, S.; Tang, C. C.; Yang, S.; Schröder, M. Direct Visualisation of Supramolecular Binding and Separation of Light Hydrocarbons in MFM-300(In). *Chem. Mat*. 2022, **34**, 5698–5705.

(9) Luo, T.; Wang, Z.; Han, X.; Chen, Y.; Iuga, D.; Lee, D.; An, B.; Xu, S.; Kang, X.; Tuna, F.; McInnes, E. J. L.; Hughes, L.; Spencer, B. F.; Schröder, M.; Yang, S. Efficient Photocatalytic Reduction of CO_2 Catalysed by the Metal-organic Framework MFM-300(Ga). *CCS Chem*. **2022**, *4*, 2560–2569.

(10) Babal, A. S.; Souza, B. E.; Möslein, A. F.; Gutiérrez, M.; Frogley, M. D.; Tan, J. C. Broadband Dielectric Behavior of an MIL-100 Metal–Organic Framework as a Function of Structural Amorphization. *ACS Appl. Electron. Mater.* **2021**, *3*, 1191–1198.

(11) Babal, A. S.; Chaudhari, A. K.; Yeung, H. H. M.; Tan, J. Guest-Tunable Dielectric Sensing Using a Single Crystal of HKUST-1. *Adv. Mater. Interfaces* **2020**, *7*, 2000408.

(12) Babal, A. S.; Donà, L.; Ryder, M. R.; Titov, K.; Chaudhari, A. K.; Zeng, Z.; Kelley, C. S.; Frogley, M. D.; Cinque, G.; Civalleri, B.; Tan, J. C. Impact of Pressure and Temperature on the Broadband Dielectric Response of the HKUST-1 Metal–Organic Framework. *J. Phys. Chem. C* **2019**, *123*, 29427–29435.

(13) Scatena, R.; Guntern, Y. T.; Macchi, P. Electron Density and Dielectric Properties of Highly Porous MOFs: Binding and Mobility of Guest Molecules in $Cu_3(BTC)_2$ and $Zn_3(BTC)_2$. *J. Am. Chem. Soc.* **2019**, *143*, 9382.

(14) Pathak, A.; Chiou, G. R.; Gade, N. R.; Usman, M.; Mendiratta, S.; Luo, T. T.; Tseng, T. W.; Chen, J. W.; Chen, F. R.; Chen, K. H.; Chen, L. C.; Lu, K. L. High-κ Samarium-Based Metal–Organic Framework for Gate Dielectric Applications. *ACS Appl. Mater. Interfaces* **2017**, *9*, 21872–21878.

(15) Mendiratta, S.; Usman, M.; Luo, T. T.; Chang, B. C.; Lee, S. F.; Lin, Y. C.; Lu, K. L. Anion-Controlled Dielectric Behavior of Homochiral Tryptophan-Based Metal–Organic Frameworks. *Cryst. Growth Des.* **2014**, *14*, 1572–1579.

(16) Balendra; A. B.; Murugavel, S.; Kanaujia, P. K.; Prakash, G. V.; Ramanan, A. Calcium and Strontium Coordination Polymers Based on Rigid and Flexible Aromatic Dicarboxylates: Synthesis, Structure, Photoluminescence and Dielectric Properties. *ChemistrySelect* **2017**, *2*, 8567–8576.

(17) Eslava, S.; Zhang, L.; Esconjauregui, S.; Yang, J.; Vanstreels, K.; Baklanov, M. R.; Saiz, E. Metal-Organic Framework ZIF-8 Films As Low-κ Dielectrics in Microelectronics. *Chem. Mater.*, **2013**, *25*, 27–33.

(18) Yu, S. S.; Yuan, G. J.; Duan, H. B. The Low Dielectric Constant and Relaxation Dielectric Behavior in Hydrogen-Bonding Metal–Organic Frameworks. *RSC Adv.* **2015**, *5*, 45213–45216.

(19) Mendiratta, S.; Usman, M.; Chang, C. C.; Lee, Y. C.; Chen, J. W.; Wu, M. K.; Lin, Y. C.; Hsu, C. P.; Lu, K. L. Zn(II)-Based Metal–Organic Framework: An Exceptionally Thermally Stable, Guest-Free Low Dielectric Material. *J. Mater. Chem. C* **2017**, *5*, 1508–1513.

(20) Li, W. J.; Liu, J.; Sun, Z. H.; Liu, T. F.; Lü, J.; Gao, S. Y.; He, C.; Cao, R.; Luo, J.-H. Integration of Metal-Organic Frameworks into an Electrochemical Dielectric Thin Film for Electronic Applications. *Nat. Commun.* **2016**, *7*, 1-8.
